# Enterovirus 71 2C Protein Inhibits NF-κB Activation by Binding to RelA(p65)

**DOI:** 10.1038/srep14302

**Published:** 2015-09-23

**Authors:** Haiwei Du, Peiqi Yin, Xiaojie Yang, Leiliang Zhang, Qi Jin, Guofeng Zhu

**Affiliations:** 1 MOH Key Laboratory of Systems Biology of Pathogens, Institute of Pathogen Biology, Chinese Academy of Medical Sciences & Peking Union Medical College, Beijing 100176, PR China; 2 Shanghai Municipal Center for Disease Control & Prevention, Shanghai 200336, China

## Abstract

Viruses evolve multiple ways to interfere with NF-κB signaling, a key regulator of innate and adaptive immunity. Enterovirus 71 (EV71) is one of primary pathogens that cause hand-foot-mouth disease. Here, we identify RelA(p65) as a novel binding partner for EV71 2C protein from yeast two-hybrid screen. By interaction with IPT domain of p65, 2C reduces the formation of heterodimer p65/p50, the predominant form of NF-κB. We also show that picornavirus 2C family proteins inhibit NF-κB activation and associate with p65 and IKKβ. Our findings provide a novel mechanism how EV71 antagonizes innate immunity.

Enterovirus 71 (EV71) is one of primary pathogens leads to hand-foot-mouth disease (HFMD) in young children and infants. HFMD caused by EV71, but not by other enteroviruses, is sometimes involved with severe neurological diseases^1^. EV71 with a positive-stranded RNA genome belongs to human enterovirus species A of the genus enterovirus within the family *Picornaviridae*^2^. The viral genome encodes a single polyprotein precursor which could be proteolytically cleaved to 4 structural and 7 non-structural proteins^2^. The nonstructural protein 2C of EV71 is composed of 329 amino acids with two functions: as an NTPase and directing replication complexes to cell membranes. EV71 2C protein has been reported to interact with host protein reticulon^3^, and this interaction is required for viral replication^3^. EV71 2C also associated with host protein coatomer, a host factor for EV71 virus^4^. Through association with IKKβ, EV71 2C inhibited TNF mediated activation of NF-κB^5^. Whether 2C targets components of NF-κB pathway other than IKKβ is not known.

NF-κB p65/p50 heterodimer, the most abundant member of NF-κB family, plays a key role in host defending virus infection^6^. Active p65/p50 dimer translocates from cytoplasm to nucleus, and promotes downstream genes transcription, such as cytokines and chemokines, which are critical for host defending virus infection through innate immunity and adaptive immunity responses^7^,^8^. Members of viruses including poxviruses, coxsackievirus, hepatitis C virus, and poliovirus have been shown to manipulate NF-κB pathway^6^,^9^,^10^,^11^,^12^,^13^,^14^.

Here, we screened EV71 2C associated proteins by yeast two-hybrid and identified p65 (RelA) as a binding partner for 2C. Moreover, we mapped the interaction between p65 and 2C. 2C could inhibit p65/p50 dimerization. We also demonstrated that picornavirus 2C family proteins could inhibit NF-κB activation and associate with p65 and IKKβ.

## Results

### Identification of host protein p65 as a binding partner for EV71 2C

**Figure 1:**
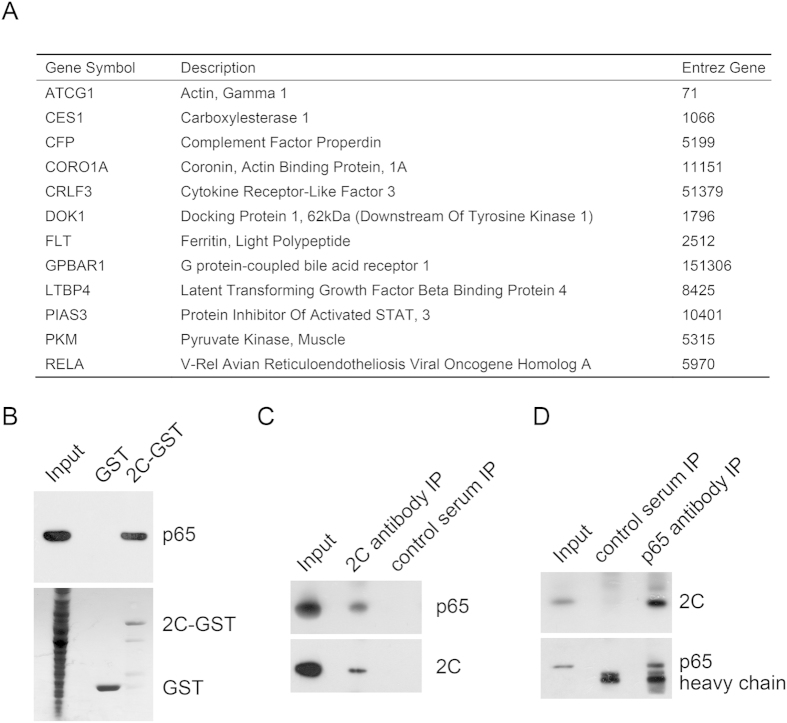
EV71 2C interacts with p65. (**A**) Candidate proteins associated with 2C from yeast two-hybrid screen. (**B**) EV71 2C interacts with p65. 2C-GST or GST immobilized on glutathione-Sepharose beads were incubated with lysates from 293T cells transfected with p65-FLAG plasmid. The bound proteins were subjected to Western blots using indicated antibodies. (**C**) Co-immunoprecipitation confirms that the EV71 2C binds to p65. RD cells were infected with EV71 for 24 h. Co-IP analysis was performed with anti-2C antibody or control serum followed by Western blot. (**D**) Co-immunoprecipitation confirms that p65 binds to EV71 2C. RD cells were infected with EV71 for 24 h. Co-IP analysis was performed with anti-p65 antibody or control serum followed by Western blot.

To explore the mechanism of 2C in the pathogenicity of EV71 infection, we screened a human Spleen Matchmaker cDNA library (Clontech, Mountain View, CA, USA) fused to the GAL4 activating domain vector using EV71 2C as a bait in AH109 yeast two-hybrid system. The positive colonies were selected on high stringency plates (lacking tryptophan, leucine, adenine and histidine) and were incubated until colonies appeared, leading to the identification of 12 host proteins that potentially interact with 2C: ATCG1, CES1, CFP, CORO1A, CRLF3, DOK1, FLT, GPBAR1, LTBP4, PIAS3, PKM, RELA (Fig. 1A). Interestingly, RelA/p65, the most abundant member of NF-κB family was found as one of the candidates to interact with 2C.

To further confirm the interaction between 2C and p65, we performed an *in vitro* GST pull-down assay with GST-fused 2C expressed in bacteria. GST-2C, but not GST, was able to pull down FLAG-p65 (Fig. 1B). To validate the interaction between the endogenous p65 and 2C in the context of EV71 infection, we performed immunoprecipitation experiment in RD cells infected with EV71 using anti-2C or anti-p65. In both cases, 2C was revealed to interact with p65 (Fig. 1C,D).

### EV71 2C protein interacted with IPT domain of p65 and inhibited p65/p50 dimerization

**Figure 2:**
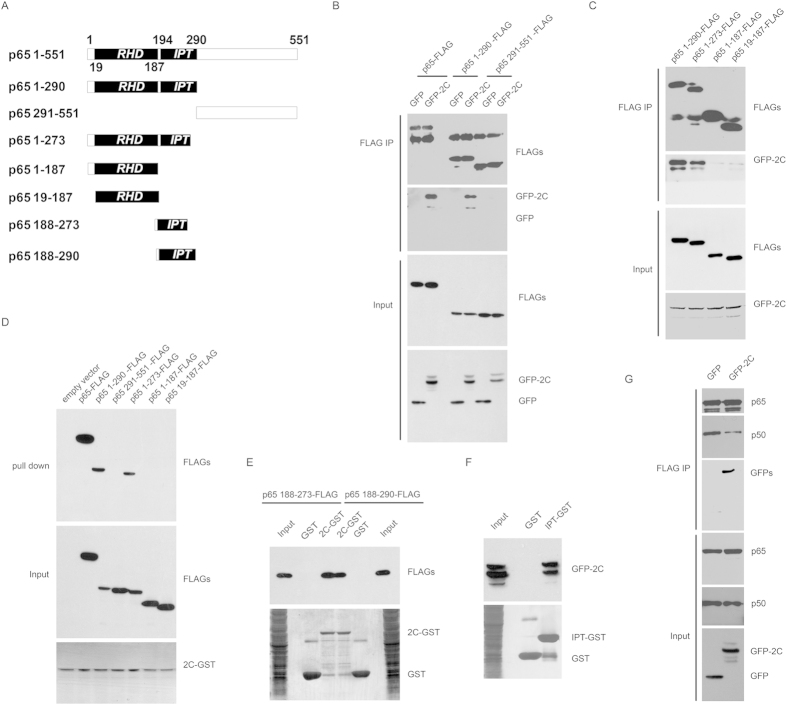
IPT domain of p65 associated with 2C. (**A**) The diagram of p65 truncations. Numbers indicated the amino acid position. (**B**) EV71 2C interacts with p65 1-290aa. 293T cells transfected with 2C and truncation constructs of p65 were analyzed by coimmunoprecipitation and Western blots using indicated antibodies. (**C**) EV71 2C interacts with 1–273 and 1–290 of p65. 293T cells transfected with 2C and truncation constructs of p65 were analyzed by coimmunoprecipitation and Western blots using indicated antibodies. (**D**) 1–273 and 1–290 of p65 interacts with 2C. 2C-GST immobilized on glutathione-Sepharose beads were incubated with lysates from 293T cells transfected with p65-FLAG or truncated p65-FLAG plasmids. The bound proteins were subjected to Western blots using indicated antibodies. (**E**) 188–273 and 188–290 of p65 interacts with 2C. 2C-GST or GST immobilized on glutathione-Sepharose beads were incubated with lysates from 293T cells transfected with indicated truncated p65-FLAG plasmids. The bound proteins were subjected to Western blots using indicated antibodies. (**F**) p65 IPT interacts with 2C. IPT-GST or GST immobilized on glutathione-Sepharose beads were incubated with lysates from 293T cells transfected with GFP-2C plasmid. The bound proteins were subjected to Western blots using indicated antibodies. (**G**) 2C inhibits p65/p50 dimerization. 293T cells transfected with p65, p50, 2C or GFP constructs were harvested and analyzed by coimmunoprecipitation and Western blots using indicated antibodies.

To map the critical region of p65 necessary for its interaction with 2C, a series of truncated p65 mutants were constructed and used for immunoprecipitation experiments (Fig. 2A). As shown in Fig. 2B, p65 1-290aa but not 291-551aa could bind with 2C, indicating that 2C specifically binds to 1-290aa of p65. Next, we generated deletion mutants including p65 1-273aa, 1-187aa and 19-187aa. As shown in Fig. 2C, 1-273aa but not 1-187aa interacted with 2C; indicating 188–273 of p65 is required for association with 2C. Similar interaction findings also were confirmed by 2C-GST pull down experiment (Fig. 2D).

To test whether 187–273 and 187–290 of p65 are sufficient to bind 2C, 2C-GST or GST immobilized on glutathione-Sepharose beads were incubated with lysates from 293T cells transfected with 187–273 or 187–290 of p65-FLAG plasmids. As shown in Fig. 2E, 187–273 and 187–290 of p65 associated with 2C-GST. IPT domain of p65 is 194–290 and we found that GST-fused IPT interacted with GFP-2C (Fig. 2F). Taken together, EV71 2C protein interacted with IPT domain of p65.

Since IPT of p65 dimerized with p50 to form p65/p50^15^, we wondered whether 2C inhibit p65/p50 dimerization. To test this hypothesis, 293T cells co-transfected with p65-FLAG/p50-Myc/2C-GFP, or p65-FLAG/p50-Myc/GFP were immnoprecipiated with anti-FLAG. As shown in Fig. 2G, the association of myc-p50 and FLAG-p65 was inhibited by 2C, suggesting that 2C could reduce p65/p50 dimer formation.

### EV71 2C targeted two components of NF-κB family, RelA and IKKβ

**Figure 3:**
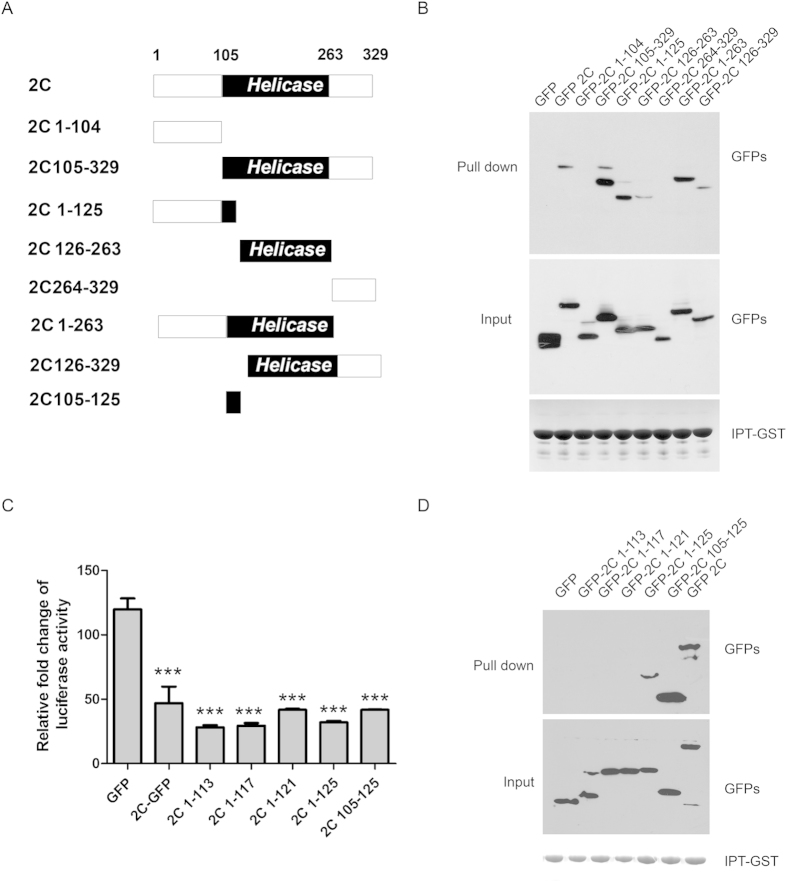
Mapping the region in 2C interacted with p65 IPT. (**A**) The diagram of 2C truncated constructs. Numbers indicated amino acid position. (**B**) IPT domain of p65 interacts with 2C 1-125aa and 126-263aa. IPT-GST immobilized on glutathione-Sepharose beads were incubated with lysates from 293T cells transfected with 2C truncated constructs. The bound proteins were subjected to Western blots using indicated antibodies. (**C**) 2C truncated forms inhibit NF-κB activation. 293T cells were transfected with pNF-κB, pRL-TK, and 2C truncated constructs for 24 hours, and then treated with TNF (10 ng/ml) for 6 hours. The cells were assayed for dual luciferase activity. Asterisks indicate significant differences between groups, data statistics were used student t-test (mean ± SD, *** indicated p < 0.001). (**D**) 2C 122-125aa is required for binding to p65 IPT domain. IPT-GST immobilized on glutathione-Sepharose beads were incubated with lysates from 293T cell transfected with 2C truncated constructs. The bound proteins were subjected to Western blots using indicated antibodies.

To map the minimal region of 2C responsible for its interaction with p65 IPT, the association of IPT with 2C mutants (Fig. 3A) was determined using *in vitro* GST pull down assay with GST-fused IPT. 1-125aa, 105–329, 126–263, 1–263, 126–329, but not 1–104, or 264–329 of p65 interacted with IPT, indicating two individual parts of p65 (1–125 and 126–263) interacted with p65 IPT domain (Fig. 3B).

Because 1–104 of 2C didn’t bind to IPT-GST while 1–125 of 2C did, we hypothesized that the IPT-associated region was narrowed down to 105–125 of 2C. Next, we constructed different truncated mutants within 1–125 including 105–125, and assessed their inhibitory effects for NF-κB activation. HEK293T cells were co-transfected with pNF-κB-luc, pRL-TK, and different regions of 2C constructs. At 24 hours post transfection, cells were treated with TNF (10 ng/ml) or mock treated for 6 hours, and assayed NF-κB activation, as described previously^11^. As shown in Fig. 3C, 105–125 of 2C inhibited NF-κB activation and associated with IPT-GST (Fig. 3D). 1–121, 1–117, 1–113 of 2C inhibited NF-κB activation (Fig. 3C) though they couldn’t bind to IPT-GST (Fig. 3D). We reasoned that those truncated forms of 2C contain 1–104, which might bind to IKKβ.

**Figure 4:**
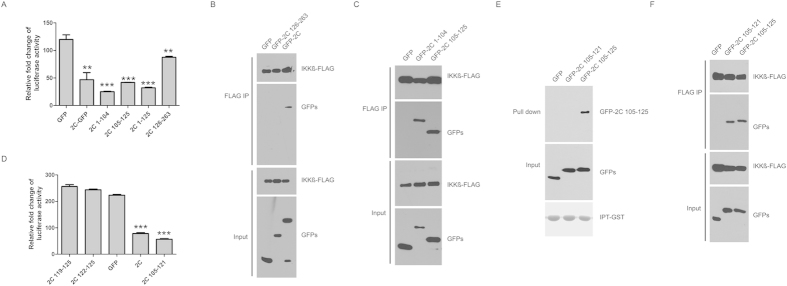
Mapping the region in 2C interacted with IKKβ. (**A**) Full length and truncated forms of 2C inhibit NF-κB activation. 293T cells transfected with pNF-κB, pRL-TK, and 2C or 2C truncated constructs for 24 hours, were treated with TNF (10 ng/ml) for 6 hours. The cells were assayed for dual luciferase activity. Asterisks indicate significant differences between groups, data statistics were used student t-test (mean ± SD, *** indicated p < 0.001). (**B**) IKKβ does not interact with 2C 126-263aa. 293T cells transfected with IKKβ and 2C or 2C truncated constructs were analyzed by coimmunoprecipitation and Western blots. (**C**) IKKβ interacts with 2C 1-104aa and 105-125aa. 293T cells transfected with IKKβ, 2C 1-104aa, 2C 105-125aa, or GFP for 36 hours were analyzed by coimmunoprecipation and Western blot using indicated antibodies. (**D**) 2C 119-125aa and 122-125aa do not inhibit NF-κB activation. 293T cells transfected with pNF-κB, pRL-TK, and 2C or 2C truncated constructs for 24 hours, were treated with TNF (10 ng/ml) for 6 hours. The cells were assayed for dual luciferase activity. Asterisks indicate significant differences between groups, data statistics were used student t-test (mean ± SD, *** indicated p < 0.001). (**E**) 2C 122-125aa is required for binding to p65 IPT domain. IPT-GST immobilized on glutathione-Sepharose beads were incubated with lysates from 293T cell transfected with 2C truncated constructs. The bound proteins were subjected to Western blots using indicated antibodies. (**F**) IKKβ interacts with 2C 105-121aa. 293T cells transfected with IKKβ and 2C 1-121aa or 2C 1-125aa were analyzed by coimmunoprecipitation and Western blots.

2C 1-125aa is known to inhibit IKKβ phosphorylation-mediated NF-κB activation through binding IKKβ^5^. Next, we assessed the inhibitory effects different 2C constructs on NF-κB activation. As shown in Fig. 4A, 1-125aa and 126–263 abrogates NF-κB activation. To test the association of IKKβ with different regions of 2C, we performed *in vivo* co-immunoprecipitation. As shown in Fig. 4B,C, 1–104 and 105–125 associated with IKKβ, while 126–263 didn’t bind IKKβ. Furthermore, we found that 105–121 of 2C inhibited NF-κB activation (Fig. 4D), while neither 119–125 nor 121–125 could. 105–121 inhibited NF-κB activation through association with IKKβ but not p65, while 105–125 could associate both IKKβ and p65 (Fig. 4E,F). These results clearly suggest that EV71 2C is actively involved in abrogating NF-κB activation by targeting two components of NF-κB family, RelA and IKKβ.

### Picornavirus 2C inhibited NF-κB activation

**Figure 5:**
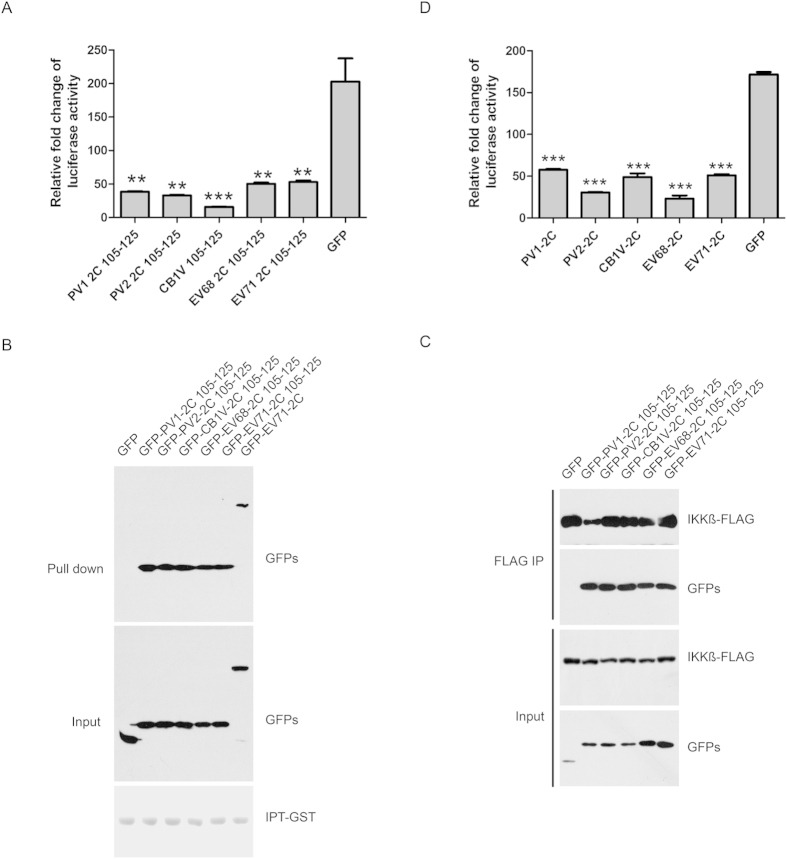
Picornavirus 2C proteins inhibit NF-κB activation. (**A**) 2C 105-125aa peptides from polivirus type I, polivirus type II, coxsackievirus B1, and EV68 inhibit NF-κB activation. 293T cells were transfected with pNF-κB, pRL-TK, and indicated 2C truncated constructs for 24 hours, and then treated with TNF (10 ng/ml) for 6 hours. The cells were harvested and assayed for dual luciferase activity. Asterisks indicated significant differences between groups, data statistics were used student t-test (mean ± SD, *** indicated p < 0.001). (**B**) 2C 105-125aa peptides from polivirus type I, polivirus type II, coxsackievirus B1, and EV68 interact with p65 IPT domain. IPT-GST immobilized on glutathione-Sepharose beads were incubated with lysates from 293T cells transfected with indicated 2C truncated plasmids. The bound proteins were subjected to Western blots using indicated antibodies. (**C**) 2C 105-125aa peptides from polivirus type I, polivirus type II, coxsackievirus B1, and EV68 interact with IKKβ. Lysates from 293T cells transfected with IKKβ and 2C 1-125aa truncated constructs were analyzed by coimmunoprecipitation and Western blots using indicated antibodies. (**D**) 2C proteins from polivirus type I, polivirus type II, coxsackievirus B1, and EV68 inhibit NF-κB activation. 293T cells were transfected with pNF-κB, pRL-TK, and indicated 2C truncated constructs for 24 hours, and then treated with TNF (10 ng/ml) for 6 hours. The cells were harvested and assayed for dual luciferase activity. Asterisks indicated significant differences between groups, data statistics were used student t-test (mean ± SD, *** indicated p < 0.001).

Next, we compared the protein sequences of picornavirus 2C family proteins and identified overall five types of 105–125 aa of picornavirus 2C family proteins. The representative viruses of 2C 105-125aa are poliovirus type I (PV1), poliovirus type II (PV2), coxsackievirus B1 (CB1), enterovirus 68 (EV68), and EV71. Interestingly, all 105-125aa of picornavirus 2C family proteins were able to inhibit NF-κB activation (Fig. 5A), suggesting that suppression of NF-κB activation by 2C is conserved across picornavirus. We also confirmed that 105-125aa of picornavirus 2C family proteins associated with both p65 IPT and IKKβ (Fig. 5B-5C). Furthermore, we generated full length 2C proteins of PV1, PV2, CB1, and EV68 and found that they all inhibited NF-κB activation (Fig. 5D).

## Discussion

In this study, we discovered that EV71 2C inhibited NF-κB activation through two different mechanisms. 105–125 and 126–263 of 2C suppressed p65/p50 dimerization probably by competing p65 IPT domain with association of p50. 1–104 and 105–121 of 2C inhibited NF-κB activation through association with IKKβ. These results have important implications in the understanding of the innate immune antagonism strategies by EV71.

Numerous studies have investigated the innate immune evasion by EV71. EV71 3C suppressed the induction of type I interferon responses through cleavage of RIG-I^16^, TLR3^17^, and IRF7^18^. EV71 2A targets IFNAR1 and MAVS to antagonize type I IFN responses and type I IFN signaling^19^,^20^. EV71-induced miR-146a targets IRAK1 and TRAF6 involved in TLR signaling and type I interferon production^21^. EV71 2C associated IKKβ and suppressed its phosphorylation to inhibit NF-κB activation^5^. Together with this, the findings presented here demonstrated that 2C targets two components of NF-κB pathway.

EV71 2C is capable to reduce p65/p50 dimer formation, which will shed important insights on other p65 associated viral proteins. The p65/p50 heterodimer is the most abundant form of the NFκB dimers. By interacting with IPT domain of p65, EV71 2C is capable to disrupt the p65/p50 heterodimer, resulting in the suppression of NF-κB activation.

Since 1–121 and 264–329 of 2C didn’t bind to IPT-GST while 105–125 and 126–329 of 2C did, the IPT-associated region was narrowed down to 122–263 of 2C. Additional investigations need to be done in the future in order to delineate the precise amino acids in 2C for interacting with p65 and IKKβ. One of strategies is to determine the structure of 122–263 of 2C with IPT. While structures of many EV71 proteins have been reported, no structure for any picornavirus 2C proteins is solved. Interaction between 2C and IPT region of p65 will provide a valuable opportunity to solve the structure of protein complex containing IPT and 2C or partial 2C.

In summary, our study provides mechanistic evidences that EV71 2C could inhibit NF-κB activation by association with p65. Two components of NF-κB pathway including p65 and IKKβ associated with 2C, suggesting that multiple mechanisms are involved for 2C to suppress the NF-κB activation. Our finding will not only further elucidate the mechanism of NF-κB activation antagonism by EV71 2C, but also advance a general understanding of picornavirus 2C proteins, as key mechanisms are likely to be conserved across all picornavirus.

## Methods

### Yeast strains, yeast plasmid, cDNA library

*S. cerevisiae* AH109 cultivated in YPD liquid (Clontech, Mountain View, CA, USA) or agar medium at 30 °C. Yeast transformant strains were cultured in lacking tryptophan, or lacking tryptophan and leucine, or lacking tryptophan, leucine, adenine, and histidine supplement medium (Clontech, Mountain View, CA, USA) at 30 °C. GAL4 binding domain vector pGBKT7 and GAL4 activating domain vector pPGADT7 were from Clontech (Mountain View, CA, USA). The vector pGBKT7 and pPGADT7 carried the tryptophan and the leucine nutritional maker for selection in yeast, respectively.

### Yeast two-hybrid screening

Competent yeast strain AH109 was transformed with bait plasmid pGBKT7-2C, according to the yeast transform system 2 manual (Clontech, Mountain View, CA, USA). After verifying that the bait plasmid pGBKT7-2C was expressed in the AH109 yeast strain and that did not activate the reporter gene, the AH109 yeast strain was transformed with human Spleen Matchmaker cDNA library (Clontech, Mountain View, CA, USA) fused to the GAL4 activating domain vector. Transformants were plated to low stringency plates (lacking tryptophan and leucine) and high stringency plates (lacking tryptophan, leucine, adenine, and histidine) until colonies appeared.

### DNA constructs

The DNA sequence encoding EV71 2C protein was cloned into the yeast plasmid pGBKT7, containing GAL4 binding domain of to generate PGBKT7-2C as bait for yeast two-hybrid screening. We transformed pEYFP-N1 vector for enzymes digestion EGFP tag and changed GFP/FLAG tags to generate pad vectors. The full-length Picornavirus 2C and 2C truncated mutations were inserted into pEGFP-C1 or padGFP vector. The full-length p65 and p65 truncations were cloned into pad-FLAG vector. EV71 2C or p65 IPT domain was cloned into the PGEX-4T-1 vector.

### Antibodies

Primary mouse antibodies included: anti-FLAG antibody (Sigma-Aldrich, St. Louis, MO, USA, catalogue No. A2220), anti-GFP were purchased from Xuheyuan (Beijing, China, catalogue No. XHY038M), IgG control (MBL, Nagoya, Japan, catalogue No. M075-3), anti-c-Myc (Santa Cruz, Dallas, TX, USA, catalogue No.), anti-p65 (Xuheyuan, Beijing, China, catalogue No. XHY076M). Primary rabbit antibodies included: anti-EV71 2C (generated against a peptide from EV71 2C [CRDRKSKVRYSVDTVVSELIREYNNRS] conjugated to keyhole limpet hemocyanin [KLH])), anti-GFP were purchased from Xuheyuan (Beijing, China, catalogue No. XHY026M). Secondary antibodies included HRP-conjugated ECL goat anti-rabbit IgG (Sigma-Aldrich, St. Louis, MO, catalogue No. A6154), HRP-conjugated ECL goat anti-mouse IgG (Sigma-Aldrich, St. Louis, MO, catalogue No. A4416). Anti-FLAG M2 affinity gel was obtained from Sigma-Aldrich (Catalogue No. A2220).

### Cell culture and cell transfection

Human embryonic kidney (HEK) 293T cells and Rhabdomyosarcoma (RD) cells were grown in DMEM supplemented with 10% heated-inactivated fetal bovine serum (Invitrogen, Carlsbad, CA, USA) and 1% penicillin-streptomycin at 37 °C in a 5% CO_2_ humidified atmosphere incubator. Cells were transfected using lipofectamine 2000 (Invitrogen, Carlsbad, CA, USA) in accordance with the manufacturer’s protocols. Enterovirus 71 (EV71) Fuyang strain (GenBank accession number: FJ439769.1) was propagated in RD cells.

### Co-Immunoprecipitations

Cells were lysed with immunoprecipitation (IP) assay buffer (50 mM Tris-HCl [pH 7.4] , 150 mM NaCl, 0.5% NP-40, 2 mM EDTA[PH8.0], 10% Glycerol) containing complete protease inhibitor cocktail (Roche, Indianapolis, IN, USA). After incubation for 30 min on ice, the lysates was centrifuged by 13000 rpm for 10 minutes. The supernatant was precleared with Protein A/G beads and then were incubated with anti-FLAG M2 affinity gel (Sigma, St. Louis, MO, USA) or antibodies on a rotator at 4 °C for 2 hours. Protein complexes captured were subjected to electrophoresis and Western blots analysis.

### Western blots

After electrophoresis, protein samples were transferred to 0.22 μm PVDF membranes (Bio-Rad, Hercules, CA, USA). The PVDF membranes were blocked with 5% non-fat dry milk (Bio-Rad, Hercules, CA, USA) and then probed with indicated primary antibodies and HRP conjugate secondary antibodies. The ECL Western Blotting Detection Kit (Applygen, Beijing, China) was used to detect chemiluminescent signals.

### Luciferase reporter assays

HEK293T cells seeded in 24-well plate were co-transfected with the plasmids pNF-κB-luc (0.1 μg/well) expressing *Firefly* luciferase, pRL-TK (0.02 μg/well) expressing *Renilla* luciferase as an internal control, and indicated expression plasmids using lipofectamine 2000 (Invitrogen, Carlsbad, CA, USA). At 24 hours post transfection, cells were treated with TNF (10 ng/ml) or mock treated for 6 hours. *Firefly* and *Renilla* luciferase activities were assessed by the dual-luciferase reporter assay system (Promega, Madison, WI, USA). To present relative fold change, we first calculated the normalized luciferase activity by divided the *Firefly* luciferase activity by *Renilla* luciferase activity. Then we divided the normalized luciferase activity with TNF by the normalized luciferase activity without TNF.

### GST pull down

GST fusion proteins were purified using GST-Sepharose (GE health, Madison, WI, USA) according to the manufacturer’s protocol. HEK293T cell lysates were extracted in lysis buffer containing 50 mM Tris, pH8.0, 100 mM NaCl, 1 mM EDTA, 1% Triton X-100 with proteinase inhibitor cocktail (Roche, Indianapolis, IN, USA). GST fusion proteins immobilized on GST-Sepharose beads were incubated with HEK293T cell lysates at 4 °C for 1 h. Beads were washed three times with lysis buffer.

## Additional Information

**How to cite this article**: Du, H. *et al.* Enterovirus 71 2C Protein Inhibits NF-κB Activation by Binding to RelA(p65). *Sci. Rep.* **5**, 14302; doi: 10.1038/srep14302 (2015).
